# Progression on Citrullination of Proteins in Gastrointestinal Cancers

**DOI:** 10.3389/fonc.2019.00015

**Published:** 2019-01-23

**Authors:** Shuzheng Song, Yingyan Yu

**Affiliations:** Department of Surgery, Ruijin Hospital, Shanghai Jiao Tong University School of Medicine, Shanghai Key Laboratory for Gastric Neoplasms, Shanghai, China

**Keywords:** citrullination, proteins, histone, PADIs, molecular targets

## Abstract

The citrullination modification (Cit) of proteins has received increasing attention in recent years. This kind of protein modification was first discovered in autoimmune diseases such as rheumatoid arthritis. The citrullination modification process is catalyzed by the peptidyl arginine deiminases (PADIs) family. A well-known citrullination of histone involves the key mechanism of neutrophil extracellular traps (NETs) of inflammation in the peripheral blood. Further studies revealed that citrullination modification of proteins also involves in carcinogenesis in human being. Citrullinated proteins disturbed the stability of proteins and caused DNA damages. There is increasing evidence that citrullinated proteins can be used as potential targets for cancer diagnosis or treatment. This review introduces the concept of citrullination modification of proteins, substrate proteins, examining methods and biological significances.

## Introduction

Proteins are the main executor of life activities. The epigenetics and post-translational modification of proteins, such as phosphorylation, acetylation, glycosylation, methylation, ubiquitination and citrullination have been found to play important roles on pathogenesis and carcinogenesis (1–3). Citrullination of proteins is a new kind of post-translational modification, which has been reported to be involved in large numbers of autoimmune diseases and cancers. This review focuses on the mechanisms, regulation, and the clinical significance of citrullinated proteins in the field of gastrointestinal diseases.

## Definition of Citrullinated Proteins

**Figure 1:**
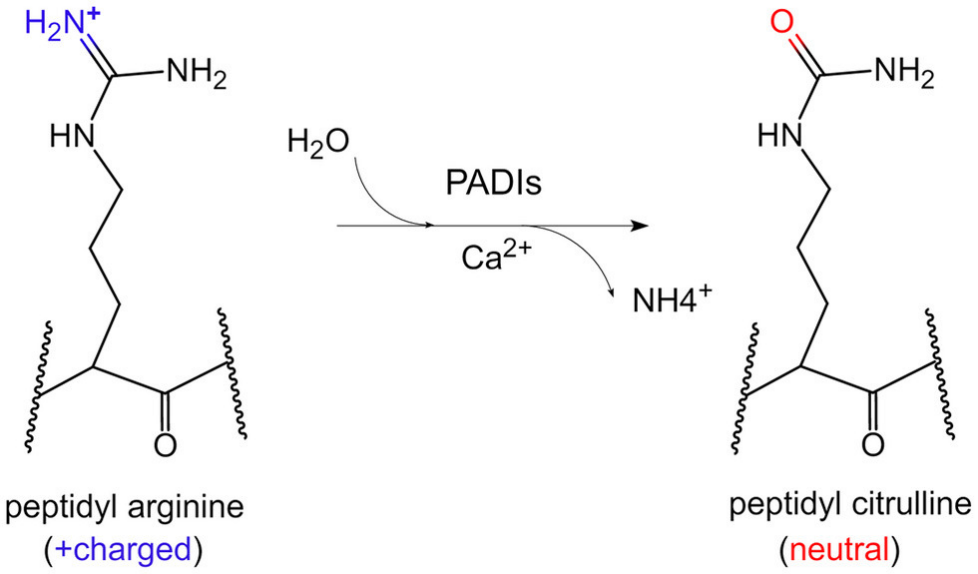
The chemical conversion process of protein citrullination. In the presence of calcium ions, PADIs could catalyze the formation of peptidyl citrulline from peptidyl arginine, which removes an amino group, accompanied by the positive charge becoming electrically neutral.

Citrullination of protein refers to the process by which the peptidyl arginine residue is converted to citrulline by a catalytic enzyme (Figure 1). Since this process is accompanied by the removal of an amino group, it is also called a peptidyl arginine deamination reaction. This chemical reaction is accompanied by a change in electrostatic charge, which may affect the folding state and function of protein, especially on histones. To date, it has been confirmed that arginine residues of dozens of proteins can undergo citrullination modifications. The substrates could be enolase, vimentin, keratin, filaggrin, serine protease inhibitors, proteases and metabolic enzymes (4). Moreover, arginine residues of histones such as H3R2/R8/R17/R26, H4R3, H2A, and H1 could be citrullinated by peptidyl arginine deiminases (PADIs) (5–8).

**Figure 2:**
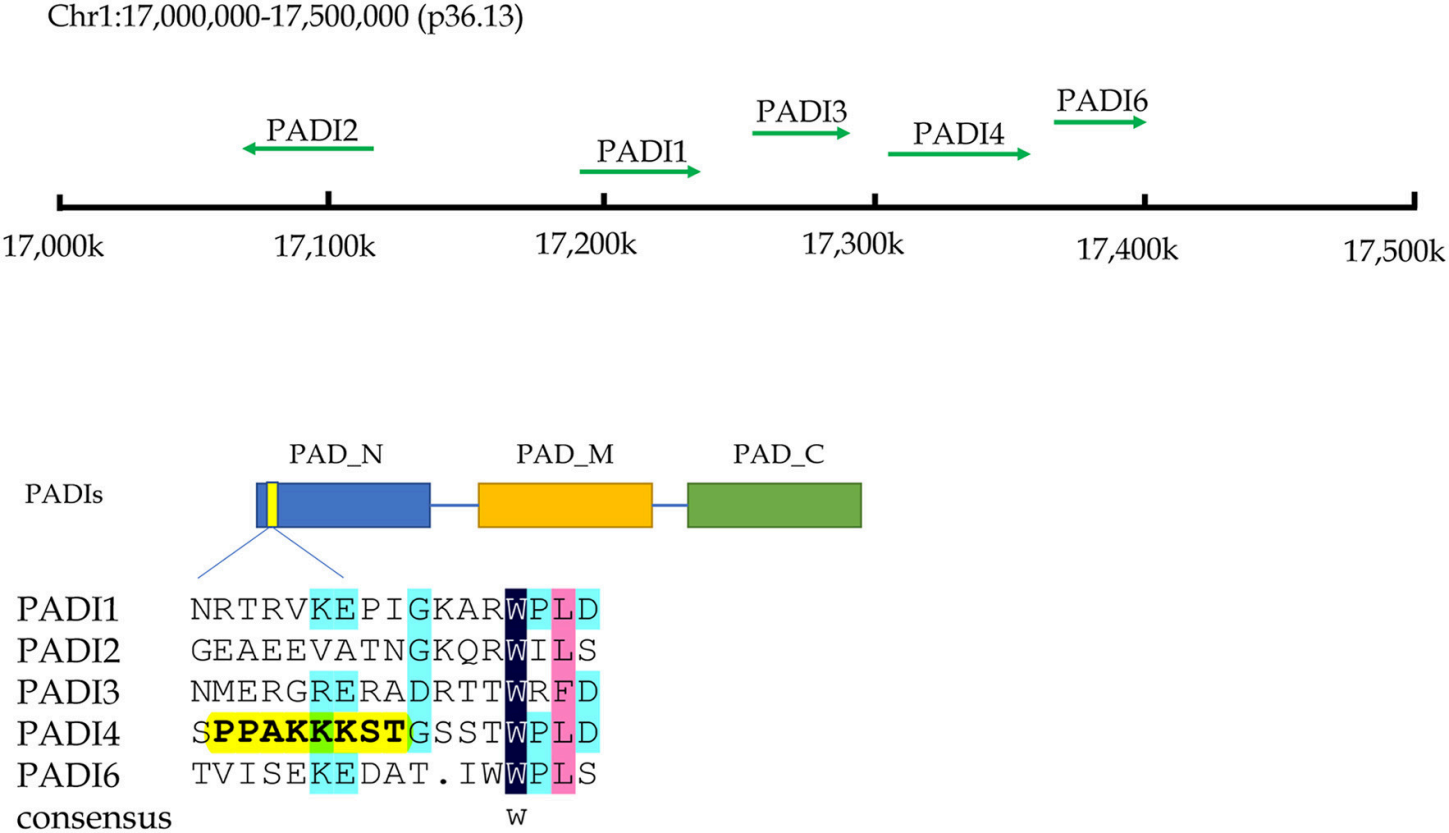
The chromosome location and structural characteristics of PADIs family. Five PADIs genes (PADI 1, 2, 3, 4, and 6) are located in the p36.13 of chromosome 1 across a region of approximately 500 k bases. PADIs members have similar structural regions, which can be divided into three sections: nitrogen zone, middle zone, and carbon end catalytic zone. Only PADI4 contains a nuclear localization signal at the nitrogen end of sequence, implying that PADI4 may play a role in the cell nucleus.

Citrullination of proteins is catalyzed by PADIs, which include five isoenzymes (PADI1-4 and PADI6) in humans. The genes of these five isozymes are located on chromosome 1p36.13. The coding regions of PADIs are about 2k in size, and consist of three parts: the nitrogen end, middle part and catalytic groups of carbon end. Regarding the subcellular localization, the PADI4 is located in the nucleus with a nuclear localization signal, while others are mainly localized in the cytosol (9) (Figure 2). PADI2 had been shown to be undergoing nuclear translocation in some cells for modifying histones (10). Therefore, citrullinated modification of histones may catalyzed by PADI4 and PADI2. The citrullination of proteins occurs in various life processes, including regulation of gene expression, immune response and protein degradation (10, 11). The citrullination of proteins is also associated with carcinogenesis in the stomach (12, 13), the large intestine (13–15), the pancreas (16), the liver (13), and so on.

## Citrullination of Non-histone Proteins

Citrullination of proteins could be induced by chemical compounds. Qu et al. reported that the antiparasitic drug nitazoxanide could induce citrullination of protein β-catenin in colorectal cancer cells via up-regulation of PADI2 enzyme. Citrullination of β-catenin resulted in the instability of the protein, and then inhibited the Wnt signaling pathway. ING4, a tumor suppressor protein, was identified as a substrate of PADI4 enzyme. Citrullination of ING4 interfered with its interaction with p53, and then decreased the tumor suppressor function in colon cancer cells (17). On the other hand, some research indicated that DNA damage induced PADI4, and then increased the citrullination of NPM1 and lamine C, which inhibited cell growth through the p53 pathway in colon cancer cells (18). Cantarino and colleagues found that down-regulation of PADI2 is an early event in the pathogenesis of colorectal cancer and is associated with poor prognosis (14). Overexpression of PADI2 inhibited cell growth and was accompanied with an increase in citrullinated protein in colon cancer cells. Overexpression of PADI2 did not increase cell apoptosis, but arrested the cell cycle in G1 phase (15). The exact effect of citrullination of proteins on cancer should be studied further.

Citrullination of proteins is not only detected in *in vitro* experiments, but also in human blood. Ordóñez et al. (19) reported that up-regulation of citrullinated antithrombin in peripheral blood of patients with rheumatoid arthritis and colorectal cancer predicted higher risk of thrombosis. Yuzhalin et al. (20) found that PADI4 could be secreted into the extracellular matrix by colorectal cancer cells, catalyzing the citrullination of proteins, thereby promoting distant metastasis of cancer cells to liver. Increased PADI4 could be found in the peripheral blood of patients with various malignancies such as gastric cancer, lung cancer, hepatocellular carcinoma, esophageal squamous cell carcinoma and breast cancer (13, 21). Until now, multiple proteins have been found as substrates of citrullination, including NF-κB p65 (22), CXCL8 (23), CXCL12 (24), E2F-1 (25), GSK3β (26), MEK1 (27), VEGFR2 (28), and so on. Obviously, citrullination of proteins involve double-sided roles in promoting both inflammation and anti-inflammation, as well as cancer promotion and inhibition.

## Citrullination of Histone PROTEINs

Citrullinated modification of histones is an epigenetic event. As introduced above, both PADI2 and PADI4 involve the citrullination process of histones in the nucleus. Recently, increased citrullinated histone H3 (H3Cit) has been considered a novel prognostic blood marker in patients with advanced cancer, due to its higher levels compared to healthy controls (29). PADI2 has been found playing an important role in mediating histone H3Cit modification, and promoting disease progression in some non-digestive cancers (30, 31). McNee et al. (32) found that PADI2 could up-regulate IL-6 expression by catalyzing H3R26Cit of bone marrow mesenchymal stem cells of multiple myeloma, which ultimately lead to chemo-resistance to bortezomib. PADI4 is another important enzyme in catalyzing the citrullination of histones. DNA damage could activate the PADI4-p53 network and catalyze histone chaperone protein, nucleophosmin (NPM1) (18). In addition, DNA damage could catalyze citrullination of the arginine 3 residue of histone H4 (H4R3cit) through the p53-PADI4 pathway in non-small cell lung cancer (33).

## Citrullination of Proteins and Immune Response

The immune system is a major weapon against cancer. Citrullination of proteins exist widely in immune-related diseases and cancers. Makrygiannakis and colleagues examined biopsy tissues from rheumatoid arthritis, myositis, tonsillitis and inflammatory bowel disease via immunohistochemistry. They found that there is a significant increase in citrullinated proteins in inflammatory tissues, compared to corresponding normal controls (34). The immune system is composed of innate immunity and acquired immunity. Neutrophils are a member of the cells of innate immunity. In process of clearing bacteria, the neutrophils secrete cell DNA, histones, and intracellular proteins to the extracellular space or circulatory system, forming so-called neutrophil extracellular traps (NETs). The citrullination of histones is involved in the process of NETs. In this process, PADI4 mediates the citrullination of histones, and results in the unwinding of DNA and subsequently excreting into the extracellular space (35–37). NETs are a self-protective mechanism against harmful bacteria. Recently, Thalin et al. found that H3Cit was significantly increased in the peripheral blood of advanced cancer patients (29). The proportion of H3Cit-positive neutrophils was increased in more serious patients. The expression level of H3Cit of serum was strongly correlated with the neutrophil activation markers, such as neutrophil elastase, myeloperoxidase and NETs-induced factors IL-6, as well as IL-8. Therefore, H3Cit is considered a useful blood biomarker for evaluating inflammatory response and prognosis in advanced cancers. Up-regulation of NETs was also identified in pancreatic ductal adenocarcinoma. The histone modification of H3Cit was proposed as a marker of NETs (16). In the pancreas, stimulating factors such as pancreatic juice could induce NETs in pancreatic ducts. Excess in NETs blocks the pancreatic duct and eventually causes pancreatitis (38).

In the cancer immunity area, the new epitopes caused by post-translational modification of proteins may provide a novel target for cancer-specific immune therapy. The condition of the cancer microenvironment including nutrient deficiency, hypoxia, redox stress and DNA damage could irritate active expression of PADIs, and catalyze production of citrullinated peptides. Increased content of citrullinated peptides may be a good target for the immune system. The cancer-specific microenvironment could induce the immune response by citrullinated peptides, and this is non-toxic and safe to the host. Carbohydrate metabolizing enzyme α-enolase is a substrate of citrullinated modification. Cook et al. (39) found that citrullination significantly induced elevation of α-enolase in Th1 immune cells, while unmodified wild-type peptides of α-enolase did not show this efficacy. Citrullinated peptides of α-enolase also induced CD4+ T activation (40, 41). The results suggested that developing tumor vaccines against citrullinated peptides of α-enolase may be a useful strategy (39). The function of citrullinated protein epitope has revealed promising utility in anti-cancer immunity.

## Detection and Biological Significance of Citrullination Modification

Citrullination modification of proteins has been reported in several fields of cancer research. Along with the progression of biomedical techniques, detection and identification of citrullinated proteins in complex biological systems becomes more feasible. Clinically, the detection of anti-cyclic citrulline antibody has been used as an assistive method for diagnosis and monitoring clinical rheumatoid arthritis (42, 43). Since the citrullination modification itself leads to 1Da mass change only, detection of the change of low abundance is still a challenging work. Phenylglyoxal (PG) could be covalently bonded with citrullinated residues specifically, and used for specific probes of labeling citrullinated proteins. The reaction could be colored by coupling dyes such as rhodamine (Rh) or biotin, and then identified by ELISA or mass spectrometry (13, 21, 29, 42, 43). By means of this technology, more and more antigens with citrullinated modification could be found, which will provide new targets for diagnosis and treatment of cancers.

In an animal experiment, Mohamed and colleagues found that nanomaterials could induce production of citrullinated protein and auto-antibodies in mice. In their study, after injection of nickel nanowires into mice, the levels of citrullinated protein and PADIs enzymes were elevated in the spleen, kidney and lymph nodes of mice, implying a systemic response to environmental materials (44). Their results suggested that safety of the nanoparticles needs to be evaluated further. Citrullination modification of proteins may be an important event for the host to recognize foreign antigens. Citrullinated proteins may be recognized as new antigens, and are promising for targeted therapy or CAR-T/NK cell-specific recognition targets.

Inhibitors of PADIs demonstrated strong potential of anti-autoimmune and anti-cancer functions *in vitro* and *in vivo*. PADI4 is the only member of the PADI family containing a nuclear localization signal, and can citrullinate many substrates including histones. PADI4 functions as a corepressor of p53 and cooperates with a histone deacetylase HDAC2 to repress the expression of tumor suppressor genes. Chlor-amidine (Cl-amidine) is a pan-PADI inhibitor that shows inhibitory effects on several members of PADIs family. However, its higher IC50 (150–200 μm) limit its preclinical exploration in cancer study and treatment (44–47). Recently, Wang and colleagues found a lead compound, YW3-56, which could activate a cohort of p53 target genes, and realize inhibitory efficacy on the mTORC1 signaling pathway, thereby disturbing autophagy and inhibiting cancerous cell growth (45). However, since the feature of a pan-PADIs inhibitor, Cl-amidine, is still be used in experimental study (48), and many new small molecule inhibitors of PADI4 are being developed by pharmacologists (49).

**Figure 3:**
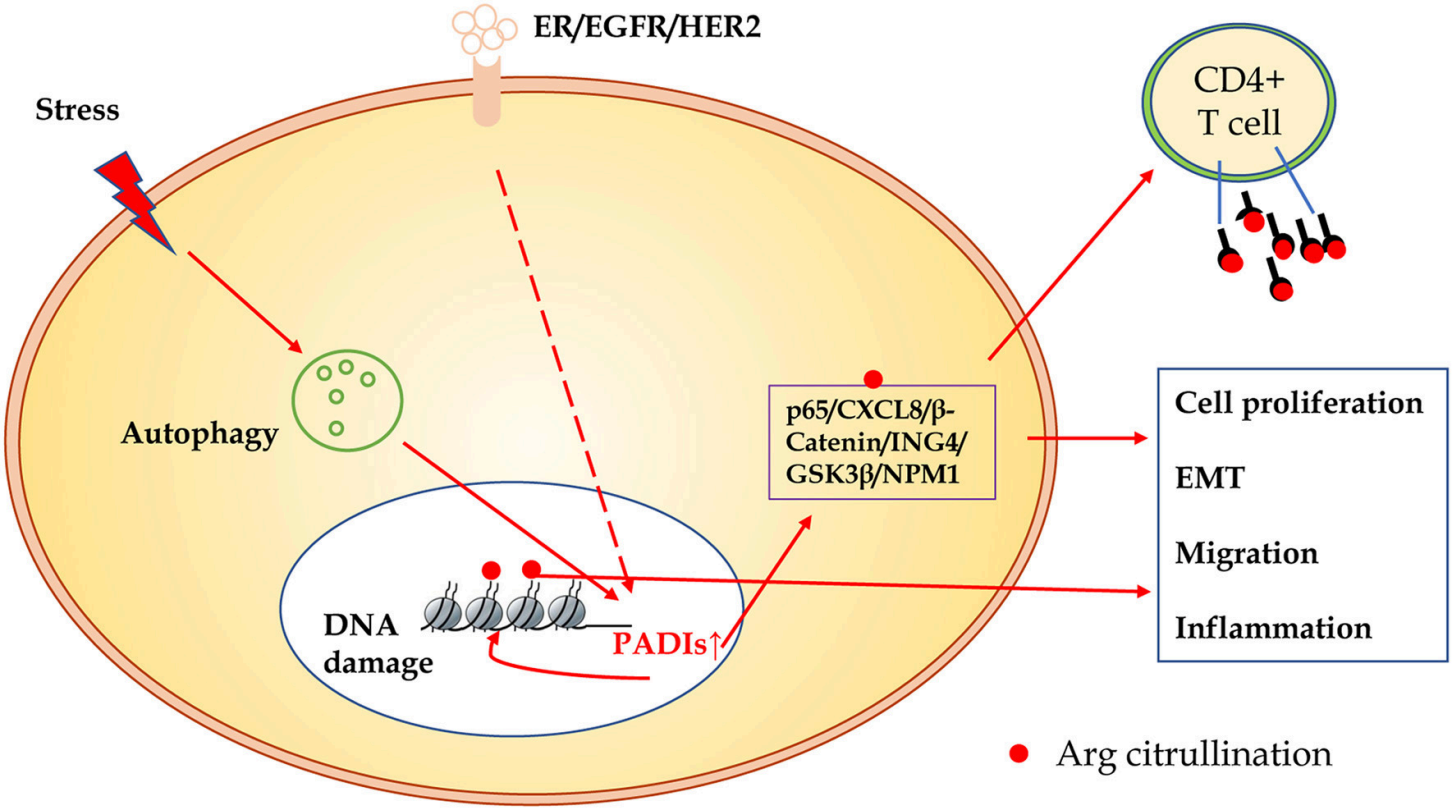
The schematic presentation of citrullinated modification of proteins and its biological significance. PADIs enzymes are activated through receptors of ER/EGFR/HER2, oxidative stress, hypoxia, and other microenvironment factors, which initiate autophagy and DNA damage. Increased PADIs catalyze citrullination modification of histones and non-histone proteins, and result in cell proliferation, epithelial-mesenchymal transition, migration, and inflammation. Citrullinated proteins as a new antigen may activate immune response of T cells or induce specific antibodies.

In summary, compared to other modification of proteins, citrullination modification is relatively novel. The exact regulatory mechanisms and biological significance in carcinogenesis are largely unclear. As shown in Figure 3, many substrates of citrullination modification are very important in life processes and development of cancers. The accurate identification of citrullination sites may help researchers to elucidate the underlying molecular mechanisms of citrullination and designing drugs for related human diseases. Several groups made efforts to predict citrullination sites by bioinformatics. Ju and Wang (50) provided a user-friendly web-server for CKSAAP_CitrSite. Zhang et al. (51) published their pioneering work of maximum-relevance-minimum-redundancy to analyze citrullination sites, and constructed classifier by random forest algorithm. We believe that in citrullination research area, bioinformatics will provide some useful insights and assistance.

## Author Contributions

SS and YY were involved in concept and design. All authors wrote, reviewed and revised the manuscript.

### Conflict of Interest Statement

The authors declare that the research was conducted in the absence of any commercial or financial relationships that could be construed as a potential conflict of interest.
